# On interpretation and task selection in studies on the effects of noise on cognitive performance

**DOI:** 10.3389/fpsyg.2014.01249

**Published:** 2014-10-30

**Authors:** Patrik Sörqvist

**Affiliations:** ^1^Department of Building, Energy, and Environmental Engineering, University of Gävle, GävleSweden; ^2^Linnaeus Centre HEAD, Swedish Institute for Disability Research, Linköping University, LinköpingSweden

**Keywords:** noise, process impurity, cognition, behavioral level of analysis, theory, method

## Abstract

This paper discusses two things researchers should consider when selecting tasks for cognitive noise studies and interpreting their findings: (a) The “process impurity” problem and (b) the propensity of sound to capture attention. Theoretical and methodological problems arise when the effects of noise on complex tasks (e.g., reading comprehension) are interpreted as reflecting an impairment of a specific cognitive process/system/skill. One reason for this is that complex tasks are, by definition, process impure (i.e., they involve several, distinct cognitive processes/systems/skills). Another reason is that sound can capture attention. When sound captures attention, the impairment to task scores is caused by an interruption, not by malfunctioning cognitive processes/systems/skills. Selecting more “process pure” tasks (e.g., the Stroop task) is not a solution to these problems. On the contrary, it introduces further problems with generalizability and representativeness. It is argued that cognitive noise researchers should employ representative noise, representative tasks (which are necessarily complex/process impure), and interpret the results on a behavioral level of analysis rather than on a cognitive level of analysis.

## INTRODUCTION

The effects of noise on cognitive performance are well documented (Jahncke et al., 2011; Clark and Sörqvist, 2012). For example, background noise impairs proofreading (Venetjoki et al., 2006), word processed writing (Keus van de Poll et al., 2014), mental arithmetic (Banbury and Berry, 1998), reading comprehension (Martin et al., 1988), and listening comprehension (Marchand et al., 2014). There has been a fundamental error in my way of thinking about these effects. There are, luckily, only a few published papers from my hand wherein this error has been made, simply because all others were rejected. And I thank the reviewers for the lectures needed. Now, as a more senior cognitive noise researcher and reviewer of manuscripts on the effects on noise on cognitive performance, I often come across both published studies and manuscripts wherein the authors—to my mind—make the very same fundamental methodological and theoretical errors as I did: They do not consider the implications of the “process impurity” problem (Surprenant and Neath, 2009) and of the propensity of sound to capture attention.

## THE PROCESS IMPURITY PROBLEM

A cognitive task, say “mental arithmetic,” whereby the participants view a sequence of numbers and report back the sum of those numbers when prompted, clearly requires the involvement of many cognitive operations. For example, short-term memory processes are needed to maintain in mind the current running total. There is also need for sustained attention (i.e., to maintain focus on the visually presented numbers). And the contents of working memory must be updated; old information that is no longer needed must be suppressed; and so on. In short, mental arithmetic—like most (if not all) cognitive tasks—is not “process pure” (Surprenant and Neath, 2009). The task score will be a measure of a conglomeration of the contribution of several sub-processes (Sörqvist et al., 2010b). The “process impurity” problem has theoretical/conceptual consequences for how effects of noise on cognitive performance in applied settings, such as schools and offices, should be interpreted.

Before turning to a discussion of these consequences, we need to understand that all cognitive tasks are process impure to some degree, especially tasks that are representative for work in schools and offices. Some tasks used in the laboratory to study basic cognitive functions are relatively process pure. For instance, a go/no-go task (i.e., responding with a key-press if and only if a specific stimulus is presented) can be considered relatively process pure as the range of possible processes or constructs the task measures is relatively limited. This is not the case with more complex tasks such as reading comprehension. Process-purity could hence be seen as a continuum from relatively pure to impure. Complex tasks are, by definition, process impure. And tasks that are representative for intellectual work, such as proofreading, mental arithmetic and word processed writing, are basically always complex.

### THE PROBLEM WITH IDENTIFYING THE BASIC COGNITIVE SKILL OR PROCESS THAT IS IMPAIRED BY NOISE WHEN USING A COMPLEX TASK

Consider studies wherein the participants are asked to study short stories, either in silence or against a background of noise, and later to answer a set of questions on the contents of the stories (e.g., Hygge et al., 2003; Boman et al., 2005; Sörqvist, 2010). This task—which may be called *delayed text recall*—is clearly not process pure. For example, the task may cover some aspect of episodic memory (e.g., retrieval of temporally and contextually tied information), but a host of cognitive processes unrelated to episodic memory (e.g., updating of working memory while reading, interpretations of the text, influences from schemata at recall) should also be measured by the task score.

A typical finding is lower task scores for texts read in the presence of background noise in comparison with texts read in silence. Based on this finding, one can be tempted to conclude that episodic memory is impaired by noise. It is, in fact, impossible to conclude that “episodic memory” is malfunction in the presence of noise, even if the participants receive lower scores in comparison with silence when they read the stories in the presence of background noise. The reason why this is impossible is because the task is not process pure. Since there is no one-to-one correspondence between the delayed text recall task and episodic memory (cf. Tulving, 2002), the task scores would drop if noise would impair (a) processes involved in the task but unrelated to episodic memory or (b) processes involved in the task which are related to episodic memory. Because of this, it may well be that episodic memory was unaffected by the noise manipulation even though the task scores where different between the noise condition and the silent condition. In other words, it is clear that the manipulation of the independent variable (i.e., presence versus absence of noise) had an effect on the dependent variable (i.e., delayed recall task scores), but the effect might have nothing to do with the functions of episodic memory.

The same conceptual problem concerns all studies wherein the effects of noise on complex cognitive tasks are studied: It is impossible to identify a basic cognitive process or function that is impaired by noise. For example, just because performance on the so-called *number updating task* is impaired by noise (Sörqvist et al., 2010a)—a task wherein the participants view a sequence of several two-digit numbers and when prompted are requested to report back the three lowest numbers presented—does not mean that the executive function called updating (i.e., the ability to exchange information in working memory) is impaired by noise. The number updating task clearly requires updating of working memory contents, but also many other sub-processes that may be selectively sensitive to noise and therefore responsible for the score decrement.

Whilst we cannot conclude, for example, that episodic memory is corrupted by noise based on the findings by Hygge et al. (2003), it is less conceptually problematic (or theory laden) to conclude that memory of written texts is impaired by noise. The latter is less problematic because memory of written texts is exactly, on a behavioral level of analysis, what is being measured (cf. Skinner, 1985; Uttal, 2007). On the behavioral level of analysis, there is no need to assume a role for specific cognitive processes or systems that may underpin the behavioral outcome. Because of this, I argue that noise effects on complex cognitive tasks should be interpreted on a behavioral level of analysis, not a cognitive level of analysis, at least when the tasks are supposed to be representative for tasks in intellectual work places.

Consider the following two examples inserted to clarify the intention with the distinction between a behavioral level of analysis and a cognitive level of analysis. *Mental arithmetic* is a skill which is underpinned by several cognitive processes (e.g., serial rehearsal). Let’s say an experiment finds an effect of noise on mental arithmetic performance (Banbury and Berry, 1998). On a behavioral level of analysis, we can conclude that noise disrupts the ability to perform mental arithmetic, but we cannot identify the exact cognitive skill that is impaired by noise and underpins the ability. *Writing* is a skill which is also underpinned by several cognitive processes (Flower and Hayes, 1981). From an experiment that finds effects of background speech on word processed writing (Sörqvist et al., 2012a), we can conclude, on a behavioral level of analysis, that the ability to produce text is impaired by noise. We cannot conclude, however, that a specific cognitive process that underpins this ability (e.g., retrieval of information from long-term memory) is impaired by noise.

### THE PROBLEM WITH SELECTIVITY

Combining different tasks is a no more successful strategy. Say that you test the effects of aircraft noise on *mental arithmetic* and *delayed text recall*, and find that aircraft noise selectively interferes with *mental arithmetic* but not with *delayed text recall*. It is not possible from investigations such as this to conclude that short-term memory but not long-term memory is impaired by aircraft noise. Even though mental arithmetic may require the continuous maintenance of the running total in short-term memory, it cannot be concluded that it is the maintenance in short-term memory that is disrupted by noise. Moreover, there is certainly a degree of overlap between the sub-processes involved in the two tasks (mental arithmetic and reading and recalling texts), an overlap that makes any conclusion about the selectivity of the effects no more than a weak speculation, on a cognitive level of analysis. It can be concluded, however, on a behavioral level of analysis, that mental arithmetic is selectively impaired by aircraft noise.

Another problem that should be mentioned in this context is that task-difficulty can modulate the effects of noise (Sörqvist et al., 2012b; Hughes et al., 2013; Sörqvist and Rönnberg, 2014). Thus, selectivity in noise effects may be caused by differences in task-difficulty (e.g., mental arithmetic may be impaired by aircraft noise whilst delayed text recall is not), not primarily because the two tasks differ in the cognitive processes or systems they entail. This further emphasizes the need to avoid speculations about differences amongst memory systems in their susceptibility to noise effects.

### IS THE USE OF MORE PROCESS PURE TASKS THE ANSWER?

Consider the Stroop task, for example, wherein the participants view color-words (e.g., RED) that are either printed in a color-congruent (e.g., red) or color-incongruent (e.g., green) ink, with the task to verbalize the color of the ink, not the meaning of the word. The difference in response time between color-congruent and color-incongruent trials is typically viewed as a measure of the costs associated with response inhibition (i.e., participants must inhibit the tendency to verbalize the meaning of the word). The Stroop task is, arguably, more process pure than complex tasks such as word processed writing.

Say that you have found an effect of noise on performance in the Stroop task (Houston, 1969). From this finding alone, it is impossible to conclude that the cognitive mechanism of response inhibition is corrupted by noise, even though the Stroop task is a relatively pure measure of response inhibition. The Stroop task is not a perfectly pure measure of response inhibition. It also measures, for example, reading speed and access to the mental lexicon. The effects of noise may, hence, operate on some other process that contributes to the task scores (or response times).

Is it at all possible to pinpoint cognitive processes that are corrupted by noise? I think the answer to this question is “yes.” However, to be able to pinpoint the exact mechanism, one must carefully manipulate the task requirements, and compare the effects of noise across different versions of the task—one version that requires the target process (e.g., serial rehearsal) and one version that does not (e.g., no requirement for serial rehearsal). An example of this is the comparison between the *short-term serial recall task* and the *missing item task*. In both tasks, the participants view sequences of visually presented words (such as six out of 7 days of the week). Hence, the material is identical in both tasks. The only difference between the two tasks is the instructions to the participants. In serial recall, the participants’ are requested to reproduce the visually presented sequence after its presentation. In the missing item task, the participants’ task is to identify the item that is missing from the set (e.g., Monday, when the other 6 days of the week has been presented). On a cognitive level of analysis, a key difference between the two tasks is that serial recall requires memory for order, whilst the missing item task does not require memory for order. The two tasks share some sub-processes (e.g., maintenance of items in short-term memory), but, arguably, they differ on the specific serial rehearsal sub-process. Years of research have shown that serial recall is disrupted by any sound that changes acoustically (Jones et al., 2010). For example, the sound sequence “k l m v r q c” is more disruptive to serial recall than the sound sequence “k k k k k k k.” In contrast, a changing state sound sequence (which does not contain a sound element that abruptly deviates from the rest of the elements in the sound stream) is no more disruptive to the missing item task than a steady state sound sequence (Jones and Macken, 1993; Hughes et al., 2007). Taken together, these findings suggest that the cognitive process of serial rehearsal, which is required in the serial recall task but not in the missing item task, is corrupted by acoustically changing task-irrelevant background sound. It is only from such careful task-requirement manipulations this conclusion is at all possible.

## ATTENTIONAL CAPTURE AND ITS CONSEQUENCES FOR INTERPRETATION

Another reason why we cannot conclude that the effects of noise on Stroop task performance is underpinned by corrupted response inhibition, is that sound can impair task performance by capturing attention (i.e., diverting the locus-of-attention away from the visual modality toward the auditory modality). Attentional capture is, arguably, the most common mechanism of auditory distraction with the capacity to interrupt any cognitive activity (Sörqvist and Rönnberg, 2014). For example, a sound sequence that contains a deviating element that stands out from the other elements in the sound stream (e.g., “k k k m k k k”) is more disruptive to the missing item task than a steady state sound stream (Hughes et al., 2007). The probable reason for this is that the deviating sound diverts the locus-of-attention away from the encoding and maintenance of the to-be-remembered items.

In the context of the Stroop task, attentional capture would prolong response time because attention is diverted away from the visual stimulus, not because response inhibition is corrupted. The same problem arises in the context of the delayed text recall task discussed above. Attentional capture, which may influence to time the participants spend on reading the text, could be responsible for the decrement in delayed text recall performance, rather than a corrupted episodic memory. The general point to be made here is: very careful sound manipulations are needed to pinpoint the mechanisms of distraction (Hughes, 2014). And even with these careful manipulations, some researchers still strongly argue that attentional capture is the only mechanism of auditory distraction (e.g., Bell et al., 2012).

## THE IMPORTANCE OF REPRESENTATIVE TASKS AND SOUND

In the context of applied noise research, is it practically fruitful—or even meaningful—to undertake the necessary task-requirement and sound manipulations needed to pinpoint the cognitive process and mechanism of distraction? The answer to this question must be based on the research question. Applied cognitive noise research often aim to study the effects of noise as they become manifest in work environments, such as schools and offices. To this end, we need to select noise that is representative for the work environment to which the results should be generalized. If the noise has to be manipulated to isolate specific mechanisms of distraction, and the noise becomes unrepresentative for the work environment in the process, then the experimental manipulation is no longer meaningful from a strict applied point of view, because the sound is (simply) not representative for the environment to which the results should be generalized. Similarly, to pinpoint the exact cognitive mechanism (or mechanisms) that are corrupted by background noise, in the context of more representative tasks such as word processed writing, requires a very careful manipulation of the task requirements. If this manipulation makes the task unrepresentative of the task office workers undertake, then the study is no longer meaningful from a strict applied point of view.

A “middle road”—for example, selecting a representative sound like road traffic noise and an unrepresentative task such as serial recall—is no more successful. For example, testing the effects of road traffic noise on serial recall would show that road traffic noise impairs performance in comparison with silence. However, the findings would say very little about how noise impairs cognitive performance on more representative tasks, such as word processed writing. Theoretically, the contribution would be limited, as we cannot know whether the observed effect is caused by corrupted serial rehearsal processes or attentional capture, let alone identify any specific cognitive capability that is impaired by noise.

The applied implications of the findings are also discouraging. Whilst serial recall is very sensitive to disruption from background sound (Jones et al., 2010), more complex tasks are not at all necessarily so sensitive. For example, word processed writing appears not to be impaired by meaningless, changing-state sound, in comparison with a silent condition (Sörqvist et al., 2012a). There are many possible reasons for this. While disruption to serial recall cannot be combated by concentrating harder on the visual modality (Hughes et al., 2013), effects of speech on more complex tasks like proofreading (Halin et al., 2014a) and delayed text recall (Halin et al., 2014b) can be combated by increased concentration. Thus, in the context of representative tasks, unlike serial recall, participants appear to be able to compensate for distraction by trying harder. Another possible reason why effects of noise are not detected in the context of more complex tasks is because they are often blunt measures of distraction. A brief reallocation of the locus-of-attention, due to attentional capture caused by background noise, has measureable consequences for serial recall, while the same attentional capture effect may not be strong enough to have measureable consequences in the context of more representative tasks. Taken together, a study of the effects of road traffic noise on serial recall say very little (both qualitatively and quantitatively) about how road traffic noise impairs representative tasks.

## CONCLUSION

When the research question is to understand and quantify the effects of noise on cognitive performance in offices, schools and other intellectual work environments, the researcher should select representative tasks and representative noise sources. Moreover, the results should be interpreted on a behavioral, not cognitive, level of analysis.

## Conflict of Interest Statement

The author declares that the research was conducted in the absence of any commercial or financial relationships that could be construed as a potential conflict of interest.
